# The Mediating and Moderating Roles of Life Skills and Cortisol in the Relationship Between Sleep Quality and Depressive Symptoms Among Chinese Adolescents With Childhood Household Dysfunction

**DOI:** 10.3389/fpsyt.2022.870349

**Published:** 2022-07-12

**Authors:** Ping Mao, Lianhua Peng, Weichao Yuwen, Dongdong Liu, Fang Yan, Yang Chen, Yixiang Long, Jonika Hash

**Affiliations:** ^1^Department of Nursing, Third Xiangya Hospital of Central South University, Changsha, China; ^2^Key Laboratory of Medical Information Research, College of Hunan Province, Central South University, Changsha, China; ^3^Xiangya School of Nursing, Central South University, Changsha, China; ^4^School of Nursing and Healthcare Leadership, University of Washington, Tacoma, WA, United States; ^5^Department of Cardiothoracic Vascular Surgery, Affiliated Hospital of Jinggangshan University, Ji’an, China; ^6^Department of Child, Family, and Population Health Nursing, School of Nursing, University of Washington, Seattle, WA, United States

**Keywords:** depressive symptoms, sleep quality, life skills, cortisol, adolescents, childhood household dysfunction

## Abstract

**Background:**

Various studies show that sleep quality, life skills, and cortisol are associated with depressive symptoms, separately. However, the relationships between sleep quality, life skills, cortisol, and depressive symptoms remain unclear. Thus, this study aims to examine the mediating or moderating roles of life skills and cortisol in the relationship between sleep quality and depressive symptoms.

**Methods:**

A retrospective cross-sectional study was performed among 212 adolescents with childhood household dysfunction (CHD) from August to October 2020 in China. We used the Pittsburgh Sleep Quality Index, the Secondary School Student Life Skills Rating Scale, and the Center for Epidemiologic Studies Depression Scale to measure sleep quality, life skills, and depressive symptoms, respectively. Additionally, 65 participants provided blood samples to assess their blood cortisol levels. Analyses included correlations, regressions, and structural equation models. Bootstrapping was performed to examine the mediation effect. Multiple regression analysis was performed to examine the moderation effect.

**Results:**

The results showed that sleep quality and life skills were significantly associated with depressive symptoms (*p* < 0.01). Life skills mediated the relationship between sleep quality and depressive symptoms. Cortisol moderated the relationship between sleep quality and depressive symptoms.

**Conclusion:**

Our findings support potential mediating and moderating roles of life skills and cortisol in the relationship between sleep quality and depressive symptoms, which suggests improving sleep quality, life skills are of great significance in the prevention and intervention of depression in adolescents with CHD, and disseminating knowledge about the high risk of developing increased depressive symptoms among adolescents with CHD with higher cortisol levels is indicated.

## Introduction

Childhood household dysfunction (CHD) refers to “children growing up in a family environment that may undermine their safety, stability, and sense of bonding” and includes experiences such as parental divorce/separation, substance abuse, severe diseases, or imprisonment (1). In China, CHD is prevalent among adolescents, with the number reaching over 0.5 million in 2018 (2). Adolescents with CHD often live in low-income areas with limited mental health resources, and they feel more loneliness and less secure compared to those with fewer CHD experiences (3). Adolescents with experiences of CHD are more likely to report depression, anxiety disorders, suicidal ideation, suicide attempt, distress, smoking, and substance abuse (4, 5). Therefore, CHD is a risk factor for various mental health problems.

Among the various negative psychological outcomes, depression is one of the most common and widely reported psychological problems among children and adolescents with CHD (6). As the subclinical stage of depression, depressive symptoms are a series of symptoms characterized by significant depressive mood, mainly including low spirits, decreased interest, physical discomfort, fatigue, and so on (7). The prevalence of depressive symptoms among adolescents is high and is still growing rapidly. The past two decades have seen an increase in elevated depressive symptoms among adolescents, with a point prevalence increasing from 24% between 2001 and 2010 to 37% between 2011 and 2020 (8). Previous studies have consistently shown that, compared with adolescents with no experience of CHD, adolescents with CHD experience depressive symptoms at a higher rate (SB = 0.045) (9). Depressive symptoms are associated with a wide range of unhealthy behaviors and negative health outcomes among adolescents. A large number of studies have shown that adolescents with depressive symptoms are at higher risk for substance abuse behaviors and suicide (10, 11). Depressive symptoms are listed as the second leading cause of death among adolescents aged between 11 and 17 (12). However, the etiology of depressive symptoms is still undefined. Besides hereditary and environmental factors (13), the effect of sleep quality on depression has received increasing attention over a long period.

Sleep quality is critical for health and development, and it is an important marker of psychological health (14). Adolescents’ poor sleep quality is related to various negative mental health outcomes, including depressive symptoms (15, 16). Recent evidence from cross-sectional studies in adolescent students shows that poor sleep quality is associated with increased depressive symptoms throughout adolescence (17). Adolescents with sleep disturbance are 2.47 times more likely to experience depressive symptoms than those without sleep problems (18). There is evidence to support the prospective role of poor sleep quality in the development of depressive symptoms in adolescents. A meta-analysis of 23 longitudinal studies among adolescents and a systematic review found sleep disturbance acts as a precursor to the development of depressive symptoms, which can predict the occurrence and outcome of depression (19, 20). Furthermore, a recent review on the association between depressive symptoms and sleep disturbance has revealed the potential mechanisms, and sleep disturbance could contribute to increased levels of inflammatory markers (e.g., IL−6, TNF, and CRP), a rapid decrease in monoamines [e.g., serotonin (5-HT), norepinephrine (NE), and dopamine], and circadian rhythm disruption (e.g., clock genes dysregulation), which are closely related to the development of depressive symptoms (20). Therefore, poor sleep quality may be a modifiable risk factor for increasing an individual’s susceptibility to depressive symptoms.

Life skills, also known as psychosocial ability, refer to the ability of individuals to adopt adaptive and positive behaviors to effectively deal with various needs and challenges in their daily life. It is considered to be a useful and effective coping ability under various stress conditions (21). WHO (21) holds that the core strategies of life skills mainly include the following: effective communication skills, decision-making, creative thinking, interpersonal relationship skills, problem-solving, critical thinking, self-awareness, empathy, and coping with stress and emotion. Numerous studies have documented the positive effects of life skills on improving mental health outcomes, especially depressive symptoms. For instance, several recent reviews have shown that life skill interventions are effective in preventing mental disorders including depressive symptoms in adolescents, especially among those in vulnerable environments [e.g., (22, 23)]. According to the transactional stress-coping model, the consequence of the interplay between stressful conditions and coping ability determines the onset of adverse mental health outcomes (24). Poor sleep quality is a stressful condition and is characterized by increased vulnerability to stressful stimuli and events, which can weaken ability to cope with stress in everyday life (25). Life skills serve as one of the most effective, useful, and universal coping abilities to help individuals better adapt to stress that may lead to psychological problems such as depressive symptoms (26). Pearlin’s stress process theory emphasizes on the process of stress can be seen as combining three major conceptual domains: the sources of stress, the mediators of stress, and the consequences of stress. In the process of the consequences of stress caused by the sources of stress (i.e., poor sleep quality), the mediators of stress are vital. Pearlin argues that coping (i.e., an individual’s ability to handle and solve the stressful condition) such as life skills plays a significant role in mediating the impact of stressful events such as poor sleep quality on depressive symptoms (27). So, we believe that life skills may mediate the relationship between sleep quality and depressive symptoms. That is, poor sleep quality acts as a chronic stressor that can be disruptive to life skills, which consequently contributes to depressive symptoms. Zhang et al. (28) found that coping style plays a mediating role in the association between sleep quality and depressive symptoms among college nursing students. However, no research has focused on life skills as the mediator between sleep quality and depressive symptoms among adolescents with CHD.

Although sleep quality and life skills are the important factors that affect depressive symptoms, the relationship between sleep quality and depressive symptoms may change because of other factors. Cortisol is a stress hormone widely recognized as an indicator for both physiological and psychological stress that affects the health status of adolescents (29). According to a recent systematic review, cortisol, as an objective and direct measure, especially cortisol in the morning, is more strongly associated with psychological distress and is possibly more important as a predictor of depressive symptoms than other subjective measures (30). Numerous literature has shown that those with higher morning waking cortisol levels have a higher risk for subsequent depression disorders (31, 32). A cross-sectional study on the potential role of cortisol dysregulation (morning plasma cortisol, 24-h urinary free cortisol, and cortisol metabolites) in depressive symptoms found that depressive symptoms in primary care subjects not consulting for their mood were associated with higher morning plasma cortisol, but not urinary cortisol or its metabolites (33). Furthermore, the cortisol awakening response can interact with stressful events (such as acute interpersonal stress) to prospectively predict depressive symptoms, which has been demonstrated in some longitudinal studies of adolescent girls (34, 35). According to the hypothesis of rapid eye movement sleep emotional homeostasis by Goldstein and Walker (36), poor sleep quality as a stressful event can interact with cortisol for the development of major depression. A recent review pointed out that the increase in morning serum cortisol caused by the dysregulation of the hypothalamus–pituitary–adrenal axis plays a positive moderating role in the relationship between poor sleep and mental health problems (37). However, whether cortisol moderates the association between sleep quality and depressive symptoms in adolescents with CHD remains uncovered.

This study aimed to explore the mediating or moderating role of life skills and cortisol in the relationship between sleep quality and depressive symptoms. The main hypotheses of this study were as follows:

**Hypothesis 1.** Life skills play the mediating roles in the relationship between sleep quality and depressive symptoms among adolescents with CHD.

**Hypothesis 2.** Cortisol moderates the relationship between sleep quality and depressive symptoms.

## Materials and Methods

### Participants and Procedures

This cross-sectional study was conducted from August to October 2020 in Hunan Province, South-Central China. Based on the Gross Domestic Product (GDP) ranking of Hunan Provincial Statistics Bureau (38), two cities were selected for the study: Changsha City (the first ranking; lies in the east of Hunan Province, mostly of Han ethnicity with high incomes) and Zhangjiajie City (the last ranking; lies in the west of Hunan Province, mostly of minority ethnicity with low incomes). Then, a random number generator computer program was used to select two counties from each city, leading to a final sampling frame of four counties.

**FIGURE 1:**
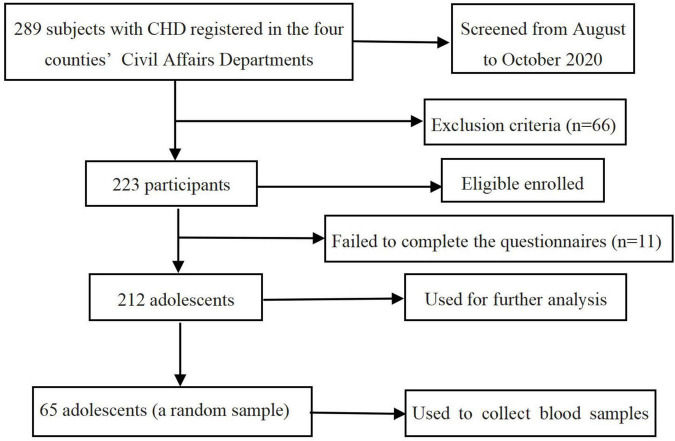
Flowchart of subject enrollment and screening.

Participants were recruited from the middle or high school students with CHD (*N* = 289) registered in the four counties’ Civil Affairs Departments. Those with mental illness and unable to communicate normally due to developmental delay were excluded from the study. In total, 223 adolescents completed the survey, among whom 212 (95.1%) were included in the final analysis (11 invalid questionnaires were excluded due to more than 30% missing items of each scale). To measure blood cortisol levels, we collected blood samples from a random sample of 65 students. The flowchart of subject enrollment and screening in this study is shown in Figure 1.

Before data collection, the research team explained the purpose of the study to potentially eligible adolescents at the schools. Adolescents who agreed to take part in this study returned home with informed consent forms. After approval was obtained from both the guardians and adolescents, the adolescents then completed the questionnaires in a single session during school hours. Investigators were present at the research sites to answer any questions raised by the participants. The blood samples were collected between 5:30 a.m. and 6:00 a.m. within 30 min of waking up the next morning in the presence of both the teacher and a trained researcher in a private room at their schools.

This study protocol and procedures were reviewed and approved by the Ethical Committee of the Xiangya School of Nursing, Central South University. Participants were provided with information regarding the study, and their informed consent was obtained.

### Sample Size Estimation

Since it was a cross-sectional study, the method of calculating the sample size by multivariable correlation study [equation: *N* = (U_1–α/2_S/δ)^2^] was used to estimate the required sample size. The pre-survey of 30 adolescents showed that the S_*depressive symptoms*_ = 0.11, S_*sleep quality*_ = 0.14, and S_*life skills*_ = 0.13. According to the allowable error δ = 0.02 (39), significance level α = 0.05, the sample size required for each variable is 116, 188, and 161, respectively. Therefore, at least 188 samples were required to be included in this study, and 212 samples were finally included.

### Measures

The research measures in this study consist of six parts:

#### Sociodemographic Characteristics

An investigator-developed questionnaire was used to obtain general demographic information such as age, gender, ethnicity, whether the participants was an only child, region of residence, annual family income, primary caregivers, and caregivers’ educational level.

#### Childhood Household Dysfunction

Childhood household dysfunction was measured using a checklist from the Chinese Ministry of Civil Affairs (40) and referred to a child whose parents had not fulfilled guardianship and parenting responsibility for more than 6 months for either one of the following: (1) incarceration, (2) severe illness determined by each locality according to the actual situation, (3) substance abuse, (4) mother remarriage, (5) death, or (6) missing. The cumulative variance contribution rate obtained *via* confirmatory factor analysis was 85.36% in this study.

#### Sleep Quality

Sleep quality during the previous month was assessed by the 19-item Chinese version of the Pittsburgh Sleep Quality Index (PSQI), which was developed for the Chinese population by Liu et al. (41). The PSQI consists of seven domain scores (subjective sleep quality; sleep latency; sleep duration; sleep efficiency; sleep disturbances; sleep medication use; and daytime dysfunction), each ranging from 0 to 3. The PSQI also yields a global score (sum of all items from the seven domains), with higher scores indicating poorer sleep quality and more severe sleep disturbances/problems. The PSQI has demonstrated good internal consistency among Chinese people (41). In this study, the PSQI also demonstrated good internal consistency with a Cronbach’s alpha of 0.87.

#### Life Skills

Life skills of adolescents in China were assessed using the Secondary School Student Life Skills Rating Scale (SSS-LSRS) developed by Zhao et al. (42). The scale includes 47 dichotomized items rated as “0” for “no” and “1” for “yes” and yields 8 major components (problem-solving ability, empathy skills, communication skills, self-esteem, sociability skills, interpersonal morality, self-efficacy, stress coping, and emotion regulation). The total score ranges from 0 to 47, with higher total scores representing better life skills. The questionnaire demonstrated good internal consistency in a previous study (43). In this study, the SSS-LSRS also demonstrated good internal consistency with a Cronbach’s alpha of 0.91.

#### Depressive Symptoms

Depressive symptoms in the past month were assessed using the Center for Epidemiologic Studies Depression Scale (CES-D) (44). The scale includes a total of 20 items and yields 4 components (depressive affect, somatic complaints, interpersonal problems, and positive affect). All items are scored on a 4-point Likert-type frequency scale, ranging from 0 (rarely or none of the time) to 3 (most or all of the time). The total score on the CES-D (sum of all items from the four components) ranges from 0 to 60, with higher scores indicating more severe depressive symptoms. According to a previous study among Chinese adolescents (45), the CES-D demonstrated good internal consistency. In this study, the CES-D also demonstrated good internal consistency with a Cronbach’s alpha of 0.94.

#### Blood Cortisol Level

To measure blood cortisol levels, we randomly selected 65 participants (35 girls and 30 boys) for a blood draw (antecubital venipuncture). A 3 ml of blood sample was collected from each participant in the early morning about 30 min (between 5:30 a.m. and 6:00 a.m.) after they woke up. Blood draws were performed in the schools by clinically experienced registered nurses. Participants were asked to avoid any food, drink, smoking, or sports 30 min prior to blood collection. After collection, all samples were placed in an incubator at 4–8°C and transported to the laboratory. Subsequently, samples were centrifuged and stored at −20°C in the laboratory until further analysis. Blood cortisol levels (nmol/L) were determined by Elecsy and Cobas e 411 Analyser (Roche Diagnostic, Switzerland), which uses the electrochemiluminescence immunoassay (ECLIA). Each sample was analyzed in duplicate and was conducted according to the manufacturer’s instructions. The analytical sensitivity of the assay was 0.05 μg/dL, with intra-assay and inter-assay coefficients of variation of <10%. For cortisol, the inter-assay variation was 2.0% and the intra-assay variation was 3.8%, which meets the EP5-A2 standard (acceptable coefficients are less than 10% for inter and intra) of CLSI (46). This means that the assay has good precision. For our analyses, we used the mean of the duplicates of each sample.

### Statistical Analysis

The data were analyzed in SPSS 26.0 and Amos 24.0 software. A *p* < 0.05 was considered statistically significant. Descriptive statistics were examined including frequencies/proportions and means/standard deviations. Pearson’s correlations were performed to examine correlations among sleep quality, life skills, and depressive symptoms. A structural equation model was used to examine the mediating effect of life skills between sleep quality and depressive symptoms. In the model, latent variables were entered for sleep quality (independent variable) and depressive symptoms (dependent variable). The mediating variable of life skills was entered as an observed variable. The bias-corrected percentile bootstrap CI method was used to calculate 95% confidence intervals (95% CIs) of the coefficients for the total, direct, and indirect effects. Coefficients were considered statistically significant if the 95% CI did not cross 0. Multiple linear regression analysis was performed to examine the moderating effect of cortisol levels between sleep quality and depressive symptoms. The regression coefficient of the interaction item of “cortisol levels” and “sleep quality” was significant, which indicates that the moderating effect of cortisol levels was significant. Age, gender, ethnicity, living region, caregivers’ education level, and the type of CHD were controlled as covariates in all analysis. To illuminate moderating effect when performing a simple slope test, we used the Johnson–Neyman method recommended by Bauer and Curran (47) to analyze the specific situation of the interaction and determine the significant interval of simple slope [i.e., the effects of the independent variable (sleep quality) on the dependent variable (depressive symptoms)] within the whole value range [(Mmin, Mmax)] of moderator (cortisol levels) and show the moderating effect of cortisol levels on the relationship between sleep quality and depressive symptoms when the level of cortisol reaches a certain value. In the moderating effect diagram, the *X*-axis represents the value of the moderator (centered and logarithmized cortisol levels), the *Y*-axis represents the slope of the effect of centered sleep quality on depressive symptoms, the solid line in the center represents the changing trend of the effect of centered sleep quality on depressive symptoms with the change of the value of moderator (centered and logarithmized cortisol levels), and the horizontal line perpendicular to the *X*-axis represents the thresholds of the moderator.

## Results

### Participant Demographics

Table 1 shows summarized demographic characteristics for the 212 adolescents with CHD in the analysis sample (mean age = 16.23 years, SD = 1.04). Of the adolescents, 54.2% were girls, 61.8% were of a minority ethnic group, 39.6% were only children, and 77.4% lived in a low-income area. Of the caregivers, 46.2% were grandparents, 42.5% were parents (one or both), and 11.3% had another (“other”) relationship with the adolescent. Regarding household dysfunction, 57.1% of participants’ fathers died and 63.2% of participants’ mothers remarried.

**TABLE 1:** Demographic characteristics of the sample.

Characteristics	*n* (%) or *M* (SD)
Age (years)	16.23 (1.04)
Gender	–
Boy	97 (45.8)
Girl	115 (54.2)
Ethnicity	–
Han ethnic	81 (38.2)
Minority ethnic	131 (61.8)
The only child in the family	–
Yes	128 (60.4)
No	84 (39.6)
Living region	–
Non-low-income area	48 (22.6)
Low-income area	164 (77.4)
Main caregivers	–
One or both parents	90 (42.5)
Grandparents	98 (46.2)
Others	24 (11.3)
Annual family income	–
< RMB 20,000	105 (49.5)
RMB 20,000–59,999	72 (34.0)
≥ RMB 60,000–99,999	35 (26.5)
Caregivers’ education level	–
Primary school or lower	105 (49.5)
Junior middle school	76 (35.8)
High school or higher	31 (14.7)
Father’s situation	–
Severe illness	36 (17.0)
Missing	29 (13.7)
Incarceration	17 (8.0)
Substance abuse	9 (4.3)
Death	121 (57.1)
Mother’s situation	–
Severe illness	23 (10.8)
Missing	39 (18.4)
Substance abuse	1 (0.5)
Remarriage	134 (63.2)
Death	15 (7.1)

*N = 212.*

### Correlations Among Sleep Quality, Life Skills, and Depressive Symptoms

There were significant correlations among sleep quality, life skills, and depressive symptoms, as shown in Table 2. Positive correlations between all dimensions of sleep quality (variables 1–7) and depressive symptoms (variables 10–13) were identified (*r*’s = 0.14–0.76, *p* < 0.05). However, life skills (variable 8) were negatively related to all dimensions of depressive symptoms (variables 10–13) (*r*’s = −0.53 to −0.38, *p* < 0.01), as well as all dimensions of sleep quality (variables 1–7; *r*’s = −0.32 to −0.19, *p* < 0.01). Additionally, cortisol levels (variable 9) were positively related to some dimensions of sleep quality (variables 3−6) (*r*’s = 0.26–0.33, *p* < 0.05) and depressive symptoms (variables 10, 12, 13) (*r*’s = 0.26–0.41, *p* < 0.05).

**TABLE 2:** Descriptive statistics and bivariate correlations among study variables.

Variable	1	2	3	4	5	6	7	8	9	10	11	12	13
1. Subjective sleep quality	–												
2. Sleep latency	0.67**	–											
3. Sleep duration	0.24**	0.21**	–										
4. Sleep efficiency	0.28**	0.25**	0.35**	–									
5. Sleep disturbance	0.75**	0.60**	0.24**	0.24**	–								
6. Sleep medication use	0.66**	0.44**	0.24**	0.31**	0.58**	–							
7. Daytime dysfunction	0.76**	0.58**	0.29**	0.28**	0.74**	0.55**	–						
8. Life skills	−0.32**	−0.29**	−0.19**	−0.19**	−0.37**	−0.22**	−0.32**	–					
9. Cortisol levels	0.19	0.04	0.30*	0.28*	0.26*	0.33**	0.16	−0.05	–				
10. Depressive affect	0.47**	0.43**	0.31**	0.27**	0.48**	0.34**	0.44**	−0.53**	0.41**	–			
11. Positive affect	0.22**	0.26**	0.17*	0.22**	0.18*	0.14*	0.22**	−0.45**	0.08	0.36**	–		
12. Somatic complaints	0.53**	0.53**	0.40**	0.24**	0.57**	0.33**	0.52**	−0.52**	0.38**	0.75**	0.26**	–	
13. Interpersonal problems	0.30**	0.20**	0.25**	0.19**	0.33**	0.23**	0.34**	−0.38**	0.26*	0.62**	0.29**	0.52**	–
Mean	1.64	1.22	0.72	0.34	1.23	0.14	1.46	25.09	470.63	5.44	3.89	5.35	0.98
SD	0.76	0.92	0.86	0.64	0.66	0.35	0.92	8.08	40.33	5.23	3.56	3.81	1.44
Range	0–3	0–3	0–3	0–3	0–3	0–1	0–3	1–47	374.50–569.10	0–23	0–12	0–15	0–6

N = 65 for correlations with cortisol. N = 212 for all other correlations.*p < 0.05, **p < 0.01.

### The Mediation of Life Skills in the Relationship Between Sleep Quality and Depressive Symptoms

**FIGURE 2:**
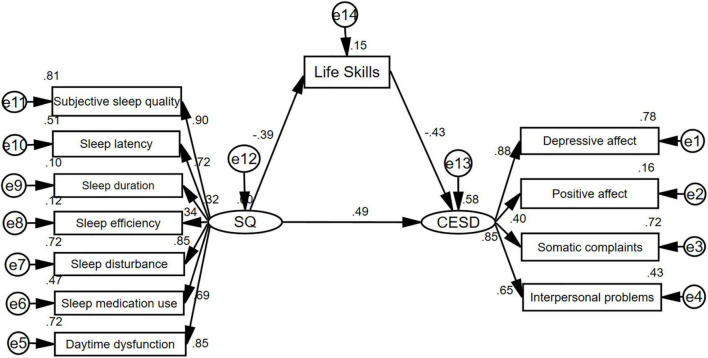
Structural model of life skills as mediator of sleep quality and depressive symptoms. SQ, Pittsburgh Sleep Quality Index; CES-D, Center for epidemiological studies depression scale.

We constructed a structural equation model for the relationships of sleep quality and life skills with depressive symptoms. The model was a good fit to the data, χ^2^ = 137.05, df = 52, χ^2^/*df* = 2.64, GFI = 0.91, IFI = 0.93, TLI = 0.91, CFI = 0.93, RMSEA = 0.079, and there were significant differences in all paths, as shown in Figure 2. The results of the bootstrap analyses further indicated that sleep quality was significantly associated with life skills (direct effect β = −0.39, 95% CI [−0.51, −0.25], *p* < 0.001) and that life skills were significantly associated with depressive symptoms (direct effect β = −0.43, 95% CI [−0.56, −0.28], *p* < 0.001). Sleep quality was significantly associated with depressive symptoms (total effect: β = 0.66, 95% CI [0.54, 0.75], *p* < 0.001; direct effect: β = 0.49, 95% CI [0.36, 0.61], *p* < 0.001). All these results suggested that life skills (β = 0.17, 95% CI [0.09, 0.25]) acted as a mediator in the relationship between sleep quality and depressive symptoms, and the mediating effect of life skills accounts for 25.8% of the total effect, as shown in Table 3.

**TABLE 3:** The total, indirect, and direct effects of sleep quality on depressive symptoms with life skills as a mediator.

Model pathways	Standardized effect estimate	Mediating effect size	Bias-corrected 95% CI	
			Lower	Upper
Total effect sleep quality → depressive symptoms	0.66		0.54	0.75
Indirect effect sleep quality → depressive symptoms	0.17	25.8%	0.09	0.25
Direct effect sleep quality → depressive symptoms	0.49	74.2%	0.36	0.61
Sleep quality → life skills	–0.39		–0.51	–0.25
Life skills → depressive symptoms	–0.43		–0.56	–0.28

### The Moderation of Cortisol Levels in the Relationship Between Sleep Quality and Depressive Symptoms

**FIGURE 3:**
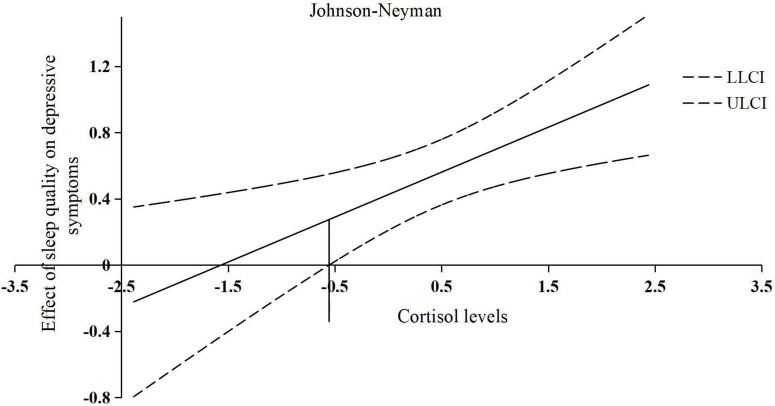
The conditional effect of centered sleep quality on depressive symptoms across the range of centered and logarithmized cortisol levels. *X*-axis: represents the value of centered and logarithmized cortisol levels; *Y*-axis: represents the slope of the effect of centered sleep quality on depressive symptoms; the solid line in the center: represents the changing trend of the effect of centered sleep quality on depressive symptoms with the change of the value of centered and logarithmized cortisol levels; the horizontal line at −0.55: represents a threshold of centered and logarithmized cortisol levels; LLCI, lower 95% CI; ULCI, upper 95% CI.

For moderation analyses with the dependent variable CES-D_*total*_, a significant interaction was found for sleep quality and cortisol levels (Δ*R*^2^ = 0.07; Δ*F*_1,61_ = 8.23; *p* = 0.006; *b* = 0.27; *t* = 2.87; *p* = 0.006), suggesting that the positive relationship between sleep quality and depressive symptoms was intensified by higher cortisol levels. However, when the centered and logarithmized cortisol values are less than the threshold of −0.55, the moderating effect of cortisol levels on the relationship between sleep quality and depressive symptoms is not significant. An overview of all moderation analyses is shown in Table 4. The interaction effects are presented in Figure 3.

**TABLE 4:** Overview of significant moderation model (including confounders) with sleep quality (SQ) as independent variables, depressive symptoms (CES-D_*total*_) as dependent variables, and cortisol levels as moderators.

Step	Variables	*b*	SE	*t*-Value	*p*-Value	95% CI	△*R*^2^	*F*-value
	Cortisol levels as a moderator							
	Constant	–0.08	0.10	–0.77	0.444	[−0.27, 0.12]		
1	Sleep quality	0.43	0.11	3.94	<0.001	[0.21, 0.64]	0.38***	18.70***
	Cortisol	0.14	0.10	1.36	0.178	[−0.06, 0.33]		
2	Sleep quality × cortisol	0.27	0.10	3.94	<0.001	[0.08, 0.46]	0.07**	16.66***

Age, gender, ethnicity, living region, caregivers’ education level, and the type of CHD were controlled in the models. b, regression coefficient; SE, standard error, ΔR^2^, R-square change. *p < 0.05, **p < 0.01, ***p < 0.001.

## Discussion

To the best of our knowledge, this is the first study to examine the role of life skills and cortisol in the relationship between sleep quality and depressive symptoms among adolescents with CHD. Our results align with prior research showing associations between sleep quality and depressive symptoms (18, 19). Our results also support the theoretical idea that life skills might mediate the association between sleep quality and depressive symptoms. Moreover, cortisol levels might moderate the relationship between sleep quality and depressive symptoms.

The finding that poor sleep quality significantly correlated with greater depressive symptoms is consistent with previous studies showing poor sleep quality as one of the most widely studied risk factors for depressive symptoms (17). Low sleep quality results in poor emotional responses such as heightened reactivity to negative emotional experiences, which further augments an individual’s susceptibility to depressive symptoms (15, 48). Furthermore, the finding that life skills are negatively associated with depressive symptoms also corroborates previous research (23). For instance, Singla et al.’s study showed that the life skill program was effective in preventing depressive symptoms among adolescents by improving emotion regulation and cognitive reappraisal (23).

In this study, life skills played a mediating role in the relationship between sleep quality and depressive symptoms. Poor sleep quality may act as a stressful event that disrupts life skills, and individuals with poor life skills may be less able to adapt to stress and adversity, which could consequently lead to depressive symptoms (26). The mediating effect of life skills in the relationship between sleep quality and depressive symptoms may be explained by Lazarus’s transactional stress-coping model (24) and Pearlin’s stress process theory (27). The transactional stress-coping model holds that cognitive appraisals serve as an essential element in an individuals’ coping process with stressful life events (24), which is closely related to sleep disturbances/problems. A systematic review has revealed that adolescents with sleep disturbances/problems (e.g., insomnia; nightmares; hypersomnia; short sleep duration; and difficulty falling asleep) might adopt negative cognitive appraisals (49). Such negative appraisals (e.g., negative situational and self-appraisals; defeat; and entrapment) may lead to negative coping such as poor life skills and finally contribute to mental health problems including depressive symptoms (27).

Furthermore, our results showed a moderating effect of cortisol levels on the association between sleep quality and depressive symptoms. In detail, cortisol levels might enhance the negative effect of sleep quality on depressive symptoms. In our study, adolescents with low sleep quality and high cortisol levels reported the highest depressive symptoms. This finding is in contrast to a previous longitudinal moderation analysis showing no significant interaction effect between sleep quality and cortisol levels (50). One explanation may be the longer periods contained in the longitudinal study design, making it difficult to observe the maintaining effects of cortisol levels in the association between sleep quality and depressive symptoms that may be affected by other time-evolving environmental factors. Further research is needed to investigate and compare the moderating effect of cortisol on the association between sleep quality and depressive symptoms.

Several limitations should be considered when interpreting the results. First, the cross-sectional design of this study cannot establish causal relationships. However, our study was based on solid theory and past research and still provides useful information on these relationships. Future longitudinal research is needed to further validate our findings. Second, the sample of this study was recruited from Hunan Province, which may not represent adolescents in other parts of China. Further research may consider using a large sample from different areas to test our study hypotheses. Third, this study only examined the relationships between sleep quality, life skills, cortisol level, and depressive symptoms among adolescents with CHD in China. Therefore, it is not clear whether the study’s findings apply to the whole adolescent population. Future studies should evaluate whether similar results could be found when evaluating different adolescent populations living in China. Finally, in our study, we only examined blood cortisol levels once in the early morning, whereas most of the studies measure cortisol levels at three different times (morning, afternoon, and night) of a day [e.g., (51)]. Cortisol releasing has a circadian rhythm (usually, the level of cortisol is highest during 6 a.m. and 8 a.m., and lowest from 10 p.m. to 2 a.m.). Compared with high levels of cortisol in the morning (such as cortisol arousal response), the increase in total cortisol production throughout the day can predict the occurrence of depressive symptoms, which may be more convincing [e.g., (52)]. However, we only collected the subjects’ serum cortisol in the morning, not multiple times a day or total cortisol throughout the day, so the analysis of the results in this study should be reviewed cautiously. Future studies may consider measuring cortisol levels multiple times a day and measuring total cortisol throughout the day to verify the accuracy of the results.

In general, this study was the first to explore the relationships among sleep quality, life skills, cortisol levels, and depressive symptoms. The results showed that sleep quality was significantly associated with depressive symptoms in adolescents. Additionally, the results supported the theoretical idea that life skills may mediate the association between sleep quality and depressive symptoms. Furthermore, the results showed that cortisol levels may moderate the relationship between sleep quality and depressive symptoms. These findings provide new evidence and potential suggestions for the prevention and intervention of adolescent depression. Families, schools, and communities should pay more attention to adolescents with poor sleep quality, low life skills, and high cortisol levels. In addition, improving life skills and sleep quality may also contribute to the improvement in adolescents’ depressive symptoms, which may have lasting beneficial effects into adulthood.

## Data Availability Statement

The raw data supporting the conclusions of this article will be made available by the authors, without undue reservation.

## Ethics Statement

The studies involving human participants were reviewed and approved by the Ethical Committee of the Xiangya School of Nursing, Central South University. Written informed consent to participate in this study was provided by the participants’ legal guardian/next of kin.

## Author Contributions

PM: conceptualization, formal analysis, writing – review and editing, and visualization. LP: visualization, data curation, formal analysis, and writing – review and editing. WY: visualization, formal analysis, and writing – review and editing. DL, FY, and YC: writing – review and editing. YL: conceptualization, formal analysis, writing – review and editing, and supervision. JH: conceptualization, visualization, formal analysis, and writing – review and editing. All authors contributed to the article and approved the final draft for submission.

## Conflict of Interest

The authors declare that the research was conducted in the absence of any commercial or financial relationships that could be construed as a potential conflict of interest.

## Publisher’s Note

All claims expressed in this article are solely those of the authors and do not necessarily represent those of their affiliated organizations, or those of the publisher, the editors and the reviewers. Any product that may be evaluated in this article, or claim that may be made by its manufacturer, is not guaranteed or endorsed by the publisher.
